# A new *Pseudophoxinus* (Teleostei, Cyprinidae) species from Southwestern Anatolia, with remarks on the distribution of the genus in western Anatolia

**DOI:** 10.3897/zookeys.320.4447

**Published:** 2013-07-31

**Authors:** Fahrettin Küçük, İskender Gülle, S. Serkan Güçlü, Yılmaz Çiftçi, Ömer Erdoğan

**Affiliations:** 1 Süleyman Demirel University, Eğirdir Fisheries Faculty, Isparta-Turkey; 2 Mehmet Akif Ersoy University, Faculty of Science and Literature, Biology Department, Burdur-Turkey; 3 Ordu University, Fatsa Marine Science Faculty, Fatsa-Ordu-Turkey

**Keywords:** Western Anatolia, Cyprinidae, Taxonomy, *Pseudophoxinus*, new species

## Abstract

*Pseudophoxinus burduricus* **sp. n.** is described from drainages of Salda and Burdur lakes, southwestern Turkey. It is distinguished from other Anatolian *Pseudophoxinus* by a combination of characters: lateral line incomplete, with 21–39 (commonly 26–37) perforated scales and 47–57+1-2 scales in lateral series; 10½–12½ scale rows between lateral line and dorsal fin origin, 3–4(5) scale rows between lateral line and the pelvic fin origin; dorsal fin commonly with 7½ branched rays; anal fin commonly with 6½ branched rays; 7–8(9) gill rakers on the first branchial arch; a faint and diffuse epidermal black stripe from eye to caudal fin base in alive and preserved individuals; mouth slightly subterminal, tip of mouth cleft on about level of lower margin of eye; snout rounded, its length greater than eye diameter. Comparison is given with all *Pseudophoxinus* species from western Anatolia.

## Introduction

According to Perea et al. (2010), there are 14 distinct clades within Leuciscinae of inner waters across the Mediterranean Region, 9 of which being represented in Anatolia. Among these, phylogenies of the genera *Petroleuciscus* and *Pseudophoxinus* are most debated; indeed even taxonomic status of some *Pseudophoxinus* species is uncertain. Two well-defined monophyletic clades represent Anatolian *Pseudophoxinus* species: first of these corresponds to Central Anatolian species complex including *Pseudophoxinus alii*, *Pseudophoxinus anatolicus*, *Pseudophoxinus antalyae*, *Pseudophoxinus battalgilae*, *Pseudophoxinus crassus*, *Pseudophoxinus elizavetae*, *Pseudophoxinus evliyae*, *Pseudophoxinus fahrettini*, *Pseudophoxinus ninae*, and a probable undescribed species; while the other includes Levantine taxa, namely *Pseudophoxinus firati*, *Pseudophoxinus kervillei*, *Pseudophoxinus zeregi* and *Pseudophoxinus zekayi* (Perea et al. 2010). Speciation in *Pseudophoxinus* is heterogenous, as mentioned by Hrbek et al. (2004) and Bogutskaya et al. (2007), and discrepancies between morphological and molecular relationships can be seen (Perea et al. 2010). Separation of the *Pseudophoxinus* taxa in Anatolia and western Asia into two groups by Bogutskaya et al. (2007), according to a comparison of morphological parameters (sensory pores, scales and their arrangement on the body, vertebral counts, and supraethmoid bone) with that of the type species *Pseudophoxinus zeregi* is another example of the high degree of variability in the genus.

Morphological and phylogenetic distinctness of *Pseudophoxinus egridiri* (Hrbek et al. 2004: 305), and its closeness to the *Pelasgus*-*Delminichthys* lineage (Perea et al. 2010) brings some doubts about monophyly of the Anatolian *Pseudophoxinus* taxa.

As stated by Hrbek et al. (2002, 2004), Anatolia is an important diversification center for the genus *Pseudophoxinus* which shows allopatric speciation especially in basins of Bey Dağları, Büyük Menderes, Tuz Lake and Lakes District. However, the complex taxonomy of the genus in these basins is still unresolved. Although including several lakes and springs of Lakes Region (Lake Salda, Karapınar Spring near Yeşilova district, Düğer Spring, Lake Bahçeözü, Sazak Spring and Kırkpınar Springs) in the distribution area of *Pseudophoxinus maeandri*, Bogutskaya (1992) mentioned that *Pseudophoxinus maeandri* populations from Upper Büyük Menderes basin (Lake Işıklı and Düden Spring near Dinar) morphologically differed from remaining populations in having larger scales and shorter lateral line, fewer lateral series scales and gill rakers on the first branchial arch, as well as fewer vertebrae.

**Figure 1.**
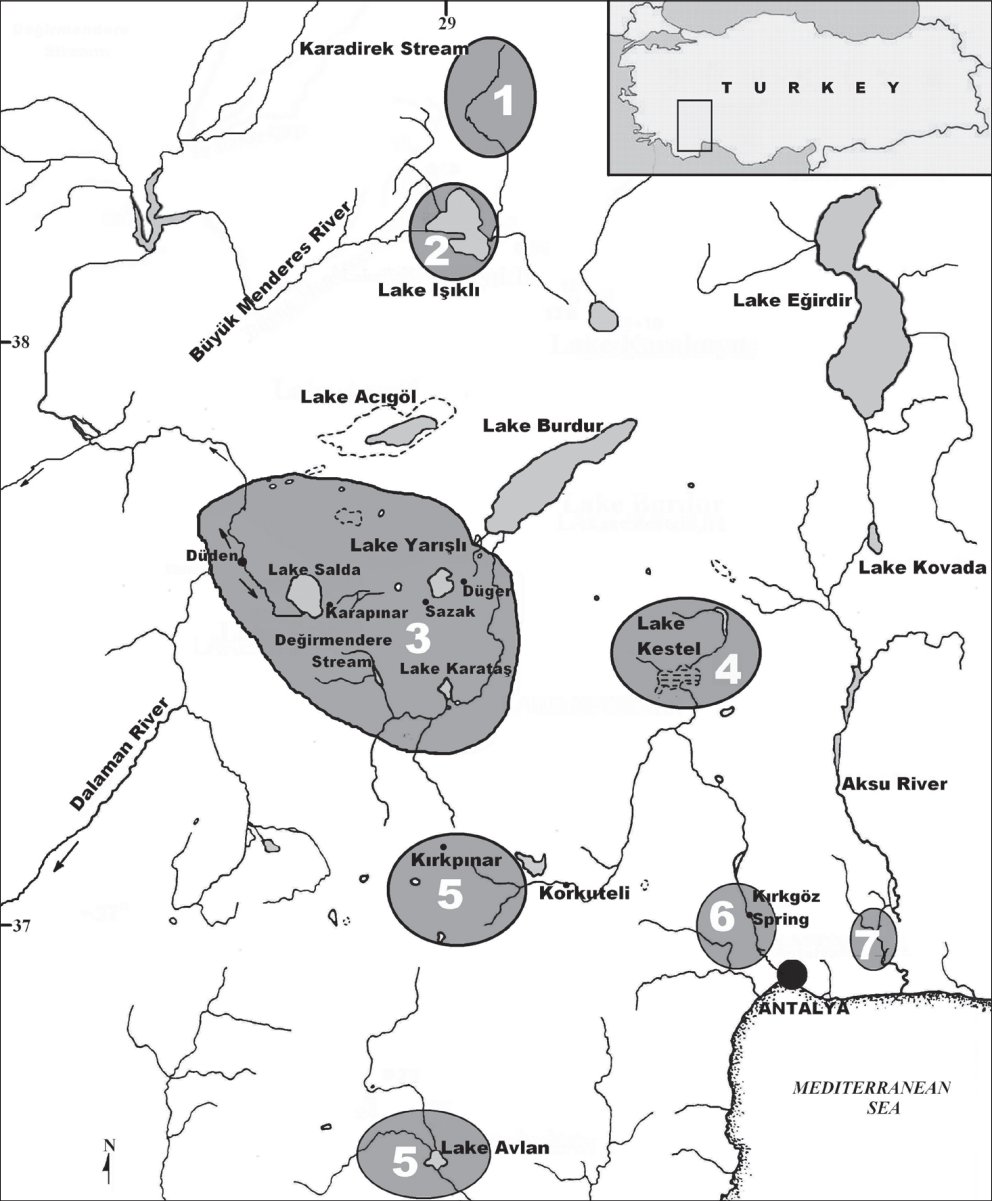
Map showing localities of *Pseudophoxinus* species in western Anatolia**1** *Pseudophoxinus maeandricus* **2** *Pseudophoxinus maeandri* **3** *Pseudophoxinus burduricus* sp. n. **4** *Pseudophoxinus ninae* **5** *Pseudophoxinus evliyae* **6** *Pseudophoxinus antalyae* **7** *Pseudophoxinus alii*

According to molecular data presented by Hrbek et al. (2004) Kırkpınar (Lake Söğüt source, Korkuteli) and Avlan source (Elmalı) populations were clearly separated from those in the basins of the lakes Salda and Burdur. Freyhof and Özuluğ (2009) identified populations of Kırkpınar as a new species, *Pseudophoxinus evliyae*, and populations of Lake Salda and Lake Burdur basin as *Pseudophoxinus ninae*. Perea et al. (2010) did not examine any material from either the type locality (Onaç Stream-Bucak) or other known localities of *Pseudophoxinus ninae* (Kestel Swamp-Bucak, Düğer Spring, Lake Karataş and Sazak Spring) and used only mitochondrial and nuclear DNA markers of Lake Salda specimens as representatives of *Pseudophoxinus ninae*. Thus, it was not possible to explain diversification of *Pseudophoxinus* in southwestern Anatolia (Fig. 1) in its entirety. Furthermore, since its description by Ladiges (1960), sufficient information on distribution and taxonomy of *Pseudophoxinus maeandricus* has not been given up to date. With regard to the above mentioned morphological and molecular data, it became apparent that the taxonomic position of *Pseudophoxinus* populations in Burdur and Salda lake basins needed clarification and this led to the present study.

## Materials and methods

Fish specimens were caught by pulsed DC electrofishing equipment and killed by over anaesthetization, preserved in 5% formalin. Material is deposited in: IFC-ESUF, Inland Fishes Collection, Eğirdir Fisheries Faculty of Süleyman Demirel University. Counts and measurements follow Kottelat and Freyhof (2007), all measurements being point to point made with a digital calliper (0.01 mm sensitive). Standard length (SL) was measured from the tip of the upper lip to the end of the hypural complex. The length of the caudal peduncle was measured from behind the base of the last anal fin ray to the end of the hypural complex, at mid-height of the caudal fin base. Lateral line scales are counted from the anteriormost scale (the first one to touch the shoulder girdle) to the posteriormost one. Scales in lateral series are counted along the midlateral line from the first one to touch the shoulder girdle to the last scale at the end of the hypural complex. Scales on the caudal fin itself are indicated by “+” (Freyhof and Özuluğ 2009). The last two branched dorsal and anal fin rays articulating on a single pterygiophore were counted as 1½. Vertebral counts were obtained from radiographs and counted as total, predorsal, abdominal and caudal vertebrae following Naseka (1996). Abdominal vertebrae were counted from the first Weberian vertebra to the one just anterior the first caudal vertebra. The first caudal vertebra is that with its haemal spine fully developed. The count of total and caudal vertebrae includes the last complex vertebra bearing hypurals. Osteological characters were examined in cleared and stained with alizarin Red-S specimens and from radiographs (Bogutskaya 1996).

The morphometric characters of the two species of *Pseudophoxinus* from Turkey were compared by Principal Component Analysis (PCA) using a covariance matrix on log–transformed measurements and counts with the software package PAST version 1.8 (Hammer et al. 2001).

**Abbreviations.** CSOsupraorbital canal; CIOinfraorbital canal; CPMpreoperculo-mandibular canal; HLlateral head length; SLstandard length. IFC-ESUFInland Fishes Collection, Eğirdir Fisheries Faculty of Süleyman Demirel University, Turkey. IUSHMIstanbul University, Science Faculty, Hydrobiology Museum, Istanbul.

## Results

### Pseudophoxinus burduricus sp. n.

href="http://zoobank.org/5734C2B8-1D58-40E4-ABD6-43A9C610B1AB

http://species-id.net/wiki/Pseudophoxinus_burduricus

Figures 2 , 3

#### Holotype.

IFC-ESUF 0427, female, 62.80 mm SL; Turkey, Burdur Prov., Değirmendere Creek, Karamanlı, Lake Burdur drainage; 37°24'18"N, 29°49'06"E, 07 November 2009, coll. F. Küçük, İ. Gülle and Ö. Erdoğan.

#### Paratypes.

IFC-ESUF 0428, 17 (11 males, 6 females), 39.90–86.69 mm SL; same as holotype.

#### Additional material.

IFC-ESUF 0236, 7, 45.31–54.40 mm SL; Burdur Prov., Salda Stream near Lake Salda; F. Küçük, M.A. Atalay, 13 June 1998. –IFC-ESUF 0289, 3, 31.98–71.56 mm SL, Burdur Prov., Düğer Spring; F. Küçük, A. Altun, M. Telli, 05 August 2006. –IFC-ESUF 0429, 5, 51.34–80.08 mm SL, Burdur Prov., Sazak Spring near Lake Yarışlı; F. Küçük, İ. Gülle, S.S. Güçlü, 13 July 2009. -IFC-ESUF 0430, 6, 41.91–55.95 mm SL, Burdur Prov., Salda Stream near Lake Salda; F. Küçük, İ. Gülle, S.S. Güçlü, 13 December 2009. –IFC-ESUF 0449, 8, 46.10-60.43 mm SL, Burdur Prov.; Salda Stream near Lake Salda; F. Küçük, İ. Gülle, 16 April 2010. –IFC-ESUF 0475, 7, 46.10–60.43 mm SL, Burdur Prov.; Dereköy Stream; F. Küçük, İ. Gülle, S.S. Güçlü. 18 May 2012.

#### Diagnosis.

*Pseudophoxinus burduricus* is distinguished from all other species of Anatolian *Pseudophoxinus* by the following unique combination of characters: head short, its length equal or slightly greater than body depth at dorsal fin origin; mouth slightly subterminal, the tip of the mouth cleft on approximately level of with lower margin of eye; snout rounded, its length greater than eye diameter; a faint and diffuse epidermal black stripe from eye to caudal fin base in alive and preserved individuals; pared fins and caudal peduncle distinctly sexual dimorphic (male with longer pelvic and pectoral fins and slenderer caudal peduncle); lateral line incomplete, with 21–37 (commonly 26–37) perforated scales and 47–57+1-2 scales in lateral series (commonly 50–55); 10½–12½ scale rows between lateral line and dorsal fin origin; 3–4 rarely 5 scale rows between lateral line and the pelvic fin origin; 7–8 (9) gill rakers on the first branchial arch; pharyngeal teeth 5–4 or 5–5, slightly serrated and hooked at tip; dorsal fin commonly with 7 (8)½ branched rays; anal fin with 6 (7)½ branched rays.

#### Description.

**Figure 2.**
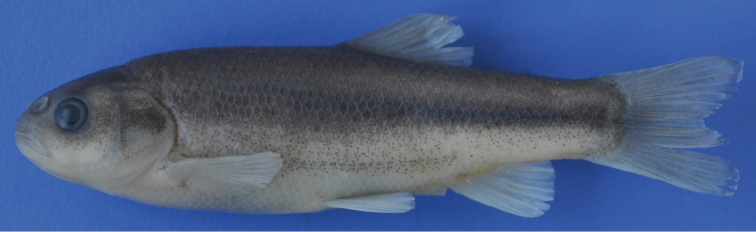
*Pseudophoxinus burduricus* sp. n. IFC-ESUF 0427,holotype, 62.80 mm SL, female; Turkey: Değirmendere Creek, Burdur.

**Figure 3.**
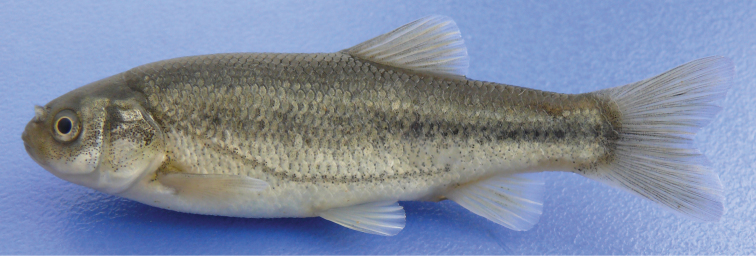
*Pseudophoxinus burduricus* sp. n. IFC-ESUF 0428,paratype, 65.82 mm SL, female; Turkey: Değirmendere Creek, Burdur.

See Figs 2–3 for general appearance and Tables 1-2 for morphometric and meristic data.

A Moderately deep-bodied, elongate and wide headed species. Dorsal profile of body slightly convex in predorsal area, ventral profile more convex than dorsal profile. Predorsal distance 52–57% SL, mean 55.0 and preanal distance 69–74% SL, mean 72.0. Head short, its length 26–28% SL, mean 26.8, approximately 1.0–1.1 times body depth at dorsal-fin origin, and its dorsal profile slightly convex on snout. Head depth at interorbital region 2.1–2.7 times eye diameter and 1.3–1.6 times interorbital distance. Mouth slightly subterminal, posterior extremity of upper jaw slightly in front of anterior margin of eye. Snout rounded, its length 27–33% HL, mean 29.6, greater than eye diameter. Caudal peduncle slightly deep, its depth 1.4–1.9, mean 1.6 times in its length.

**Table 1.** Morphometry of *Pseudophoxinus burduricus* sp. n. (holotype IFC-ESUF 427, paratypes IFC-ESUF 428, n=17) and *Pseudophoxinus ninae* (IFC-ESUF 263, n=15).

	***Pseudophoxinus burduricus***		***Pseudophoxinus ninae***
	**Holotype**	**Paratypes**	
**In percent of standard length**			
Head length	26.9	25.7–27.8 (26.8) ±0.6	27.1–30.7 (28.6) ±1.0
Body depth of dorsal fin origin	26.6	24.1–27.1 (25.7) ±0.7	27.8–32.1 (29.3) ±1.2
Predorsal distance	56.6	51.6–57.4 (55.0) ±1.7	56.6–60.9 (58.0) ±1.1
Prepelvic distance	52.1	50.4–54.7 (52.6) ±1.5	53.9–56.7 (55.7) ±0.9
Preanal distance	72.4	68.7–74.0 (72.0) ±1.5	72.6–76.5 (74.5) ±1.1
Distance between pectoral and anal-fin origins	48.4	43.0–51.1 (48.1) ±2.5	47.3–51.3 (49.0) ±1.1
Distance between pectoral and pelvic-fin origins	26.7	24.6–31.2 (27.7) ±1.8	27.3–30.6 (29.0) ±0.8
Distance between pelvic and anal-fin origins	21.8	17.7–22.8 (20.0) ±1.6	18.3–22.2 (20.0) ±1.4
Dorsal fin depth	20.8	16.9–22.1 (20.1) ±1.7	16.9–21.7 (19.6) ±1.5
Anal fin length	18.3	15.2–20.6 (17.8) ±1.5	14.1–20.1 (17.0) ±1.7
Pectoral fin length	19.4	17.6–25.0 (21.3) ±2.1	17.7–22.1 (19.8) ±1.4
Pelvic fin length	15.6	13.7–19.1 (16.3) ±1.6	13.6–18.6 (15.7) ±1.4
Caudal peduncle length	20.5	17.5–23.0 (20.1) ±1.4	17.3–21.3 (19.0) ±1.3
Caudal peduncle depth	13.1	11.4–14.9 (12.6) ±0.9	12.7–14.9 (13.5) ±0.6
**In percent of head length**			
Snout length	29.4	26.5–32.9 (29.6) ±1.9	22.0–28.9 (25.4) ±1.8
Eye diameter	22.6	22.0–25.9 (24.5) ±1.4	20.5–23.3 (21.9) ±0.9
Interorbital distance	38.3	34.6–40.5 (38.2) ±1.6	30.6–36.3 (33.6) ±1.9
Head width at nape	59.8	55.3–61.7 (58.7) ±1.6	53.8–63.2 (58.0) ±2.8
Head depth at interorbital region	60.6	50.2–62.2 (56.7) ±3.3	49.7–58.2 (53.7) ±2.6
Head depth at nape	79.4	76.5–85.3 (80.7) ±2.8	70.8–80.7 (74.9) ±1.5
Operculum depth	42.0	40.0–48.8 (43.4) ±2.3	36.5–46.6 (40.1) ±2.6
Lower jaw length	36.0	32.6–38.6 (35.5) ±1.2	31.3–35.6 (33.5) ±2.1

**Table 2.** Meristic features of the western Anatolian *Pseudophoxinus* species.

**Species**	**Lateral series**	**Lateral line**	**Pharyngeal teeth**	**Total vertebrae**	**Abdominal vertebrae**	**Caudal vertebrae**
*Pseudophoxinus alii*	41–44	38–41	5–5	37–39	21–23	16–17
*Pseudophoxinus antalyae*	52–64	42–59	5–5	37–39	20–22	16–17
*Pseudophoxinus anatolicus* (*)	93–109	78–93	5–5	41–42	23–24	17–18
*Pseudophoxinus battalgilae* (**)	53–61	53–60	5–5	37–38(39)	20–21	17–18
*Pseudophoxinus burduricus* sp. n.	47–57	21–39	5–4(5)	36–39	21–22	15–17
*Pseudophoxinus crassus* (*)	65–78	62–73	5–5	40	22	18
*Pseudophoxinus elizavetea* (**)	60–68	33–60	5–5	36–37 (38)	22	14–16
*Pseudophoxinus evliyae*	52–66	16–30	5–4	36–37	21–22	14–16
*Pseudophoxinus hittitorum*	83–96	83–94	5–5	39	21–22	17–18
*Pseudophoxinus maeandri*	41–45	19–27	5–4	35–36	19–20	15–16
*Pseudophoxinus maeandricus* (**)	66–67	58–65	5–5	36–37	20–21	16
*Pseudophoxinus ninae*	46–53	10–32	5–4	36	21	15

(*) from Atalay 2005

(**) Bogutskaya et al. 2007

Lateral line incomplete, usually reaching above anal fin origin, 32 perforated scales in the holotype (26–37 in paratypes), 47–57+1-2 scales in lateral series. Dorsal fin with 3 simple and 7½ (n=18, in one specimen 8½) branched rays, outer margin slightly convex. Anal fin with 3 simple and 6 (15)½ or 7 (3)½ branched rays, outer margin slightly convex. Pectoral fin with 13–14 branched rays, outer margin straight or slightly convex. Pelvic-fin with 7 branched rays. Caudal fin forked, lobes rounded. There is no pelvic axillary lobe and keel between posterior pelvic fin base and anus. Pharyngeal teeth 5–4 or 5–5, slightly serrated, hooked at tip (developed on the left side). Gill rakers short and thick, with 7–8 (9) in outer side of first gill arch. Preoperculo-mandibular (CPM) and infraorbital (CIO) sensory canals disconnected, CSO with 10–12 pores, CIO with 14–19 pores, CPM with 14–18 pores, total vertebrae 36–39, 21–22 abdominal and 15–17 caudal vertebrae, vertebral formulae: 36–39:21–22+15–17.

**Sexual dimorphism.** In Değirmendere population, there is no tubercules on snout and head in males, which have longer pelvic and pectoral fins and slender caudal peduncles than females. In Lake Salda population, on the other hand, tubercules present in males on entire body (concentrated on operculum) and all fins except for the caudal fin.

**Coloration.** Body silvery, dorsal light brown or olive green and scales irregular with small epidermal spots in specimenslarger than50 mm SL. There is a faint epidermal black or violet (in Sazak population) stripe along lateral midline from eye to caudal fin base in alive. The dark stripe indistinct or slightly distinct in anterior part of body but distinct in posterior part of body in preserved specimens. Lateral line scales with small brown to black spots above and below pores in some individuals. Fin membranes whitish or light grey, rays with black-spotted. In individuals smaller than 50 mm SL: body silvery, dorsal dusty grey, ventral pearl grey.

#### Distribution.

*Pseudophoxinus burduricus* is known only from the lakes and their sources in Lake Burdur Endorheic Basin: Değirmendere Creek, Lake Karataş, Düğer and Sazak (or Kümbet) springs, Dereköy Stream, Lake Salda and Salda Stream (Fig. 1). Değirmendere Creek is a 5 km long creek flowing into the Karamanlı reservoir, which is connected with artificial reservoir Lake Karataş. Düğer spring is a source of Lake Burdur, while Sazak Spring is a source of Lake Yarışlı. Dereköy Stream is an approximately 5 km long rivulet formerly draining into Çorak (or Akgöl) Lake which, due to a small reservoir constructed in 1970, no more can reach the lake. The new species can be encountered in the shallow parts of the reservoir and the small stream flowing into it.

Other species present were: *Chondrostoma fahirae* (Ladiges, 1960), *Oxynoemacheilus anatolicus* Erk’akan, Özeren & Nalbant, 2008 and *Oncorhynchus mykiss* (Walbaum, 1792) (an escape from fish farms in Karamanlı reservoir)in Değirmendere, in Düğer Spring only *Oxynoemacheilus anatolicus* and in Salda Stream only *Aphanius splendens* (Kosswig & Sözer 1945).

#### Etymology.

The species is named after the Burdur Province where the type locality is located.

## Discussion

Hrbek et al. (2004) stated that monophly of Anatolian *Pseudophoxinus* taxa was not well supported and these represented 6 distinct clades. Of these, Lakes Region populations (Lakes Salda and Karataş, Düğer Spring, Karapınar) identified as *Pseudophoxinus maeandri* formed Clade IV, while the Avlan and Kırkpınar populations identified as *Pseudophoxinus fahirae* (now *Pseudophoxinus evliyae*) formed a separate clade (Clade VI). Perea et al. (2010) mentioned of two well defined monophyletic groups within Anatolian *Pseudophoxinus* taxa, though not discussion interrelationships of these two. A phylogenetic tree based on the cytb gene sequence, Hrbek et al. (2004: 299) showed presence of two different clade; (I) Lake Avlan and Kırkpınar (Lake Söğüt source) populations (*Pseudophoxinus evliyae*) and (II) populations from Burdur and Salda basins.

In this study, we also morphologically compared the new species with *Pseudophoxinus ninae* (the Onaç Stream and Kestel Swamp), *Pseudophoxinus evliyae* (dried Lake Söğüt [Kırkpınar Village, Korkuteli] and source of Lake Avlan [Elmalı]), *Pseudophoxinus maeandri* [Lake Işıklı], *Pseudophoxinus maeandricus* (Karadirek Stream [Upper Büyük Menderes basin]), *Pseudophoxinus alii* (Köprüçay and Ilıca Stream), *Pseudophoxinus battalgilae* (Manavgat River basin, Lake Akgöl [Ereğli], Lake Çavuşcu [Ilgın] and Lake Suğla [Seydişehir]), *Pseudophoxinus fahrettini* (Köprüçay River basin [Bağıllı Village and Değirmenözü Stream]), *Pseudophoxinus antalyae* (Kırkgöz Spring, Karamanlı Stream and tributaries of Düden canal in Antalya), and *Pseudophoxinus elizavetae* (Sultansazlığı [Kayseri]).

**Figure 4.**
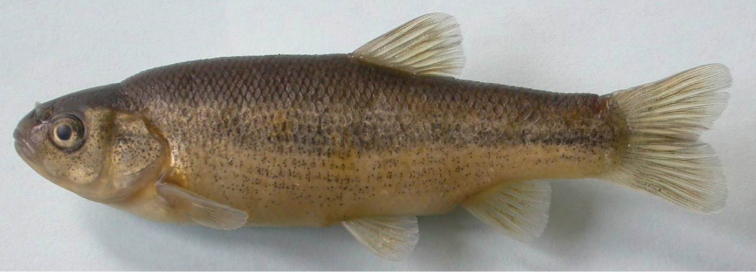
*Pseudophoxinus ninae* IFC-ESUF 0263, 66.37 mm SL; Turkey: Pınargözü Spring-Bucak.

**Figure 5.**
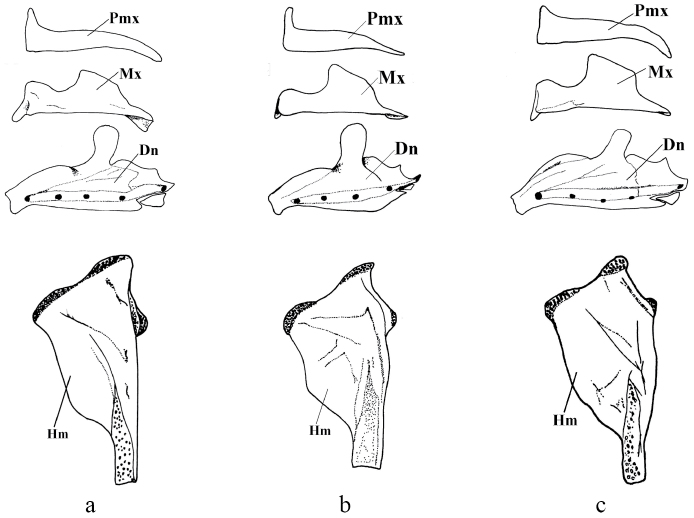
Left jaws and hyomandibular bones of *Pseudophoxinus burduricus* sp. n. (**a**) *Pseudophoxinus ninae* (**b**)and *Pseudophoxinus maeandri* (**c**) (Pmx: premaxilla, Mx: maxilla, Dn: Dentale, Hm: Hyomandibulare)

**Figure 6.**
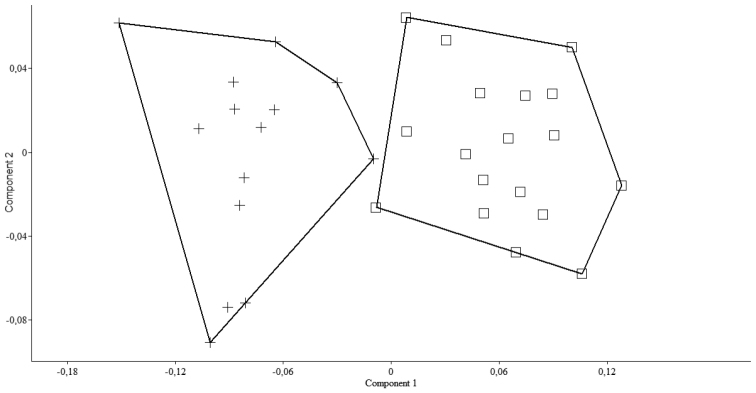
A scatter plot of the scores of the first two principal components (PC I, PC II) for 32 specimens of the two *Pseudophoxinus* species (*Pseudophoxinus burduricus* sp. n. (+) and *Pseudophoxinus ninae* (□), based on 18 morphometric characters.

*Pseudophoxinus burduricus* is most similar to *Pseudophoxinus ninae* (Fig. 4). Itis distinguished from *Pseudophoxinus ninae* by having fewer branched pelvic fin rays (7, vs. 8 or 9), slightly fewer gill rakers in outer side of the first gill arch (7–8, rarely 9, vs. 8–9) and a longer caudal peduncle (caudal peduncle length 1.4–1.9 times caudal peduncle, vs. 1.3–1.4). It further differs from *Pseudophoxinus ninae* by having a shorter predorsal distance (52–57% SL, mean 55.0, vs. 57–61, mean 58.0), a shorter preanal distance (69–74% SL, mean 72.0, vs. 73–77, mean 74.5), a somewhat shorter head (head length 26–28 % SL, mean 26.8, vs. 27–31, mean 28.6), and narrower and relatively deeper hyomandibular bone (vs. wide and shallow) (see Figs 5a, b, c). Also *Pseudophoxinus burduricus* and *Pseudophoxinus ninae* were compared by Principal Component Analysis (PCA). The PCA was performed in using 18 morphometric characters of the two *Pseudophoxinus* species. The PCA clearly separated *Pseudophoxinus burduricus* from *Pseudophoxinus ninae* (Fig. 6). Variables loading on the first metric PC I–II are given in Table 3.

**Table 3.** Character loading on principal components I–II for 18 measurements taken on 32 specimen of two *Pseudophoxinus* species (*Pseudophoxinus burduricus* sp. n. and *Pseudophoxinus ninae*).

**Morphometric features**		
**In percent of standard length**	PC I	PC II
Head length	0.196	-0.103
Body depth of dorsal-fin origin	0.335	0.222
Predorsal length	0.140	0.119
Prepelvic length	0.147	0.145
Preanal length	0.083	0.156
Dist. from pectoral-fin origin to anal fin	0.060	0.331
Dist. from pectoral-fin origin to pelvic fin	0.140	0.396
Dist. from pelvic-fin origin to anal fin	0.012	0.368
Length of caudal peduncle	-0.300	-0.383
Depth of caudal peduncle	0.180	0.112
**In percent of head length**		
Snout length	-0.445	0.322
Eye diameter	-0.285	-0.118
Interorbital distance	-0.420	0.166
Head width (at operculum)	-0.054	0.300
Head depth (at interorbital region)	-0.202	0.080
Operculum depth	-0.290	0.155
Head depth operculum	-0.208	0.164
Length of lower jaw	-0.172	0.164

*Pseudophoxinus burduricus* is distinguished from *Pseudophoxinus evliyae* by having fewer scales in lateral series (47–57+1-2, vs. 54–64+1-2), fewer branched pelvic fin rays (7, vs. 8), fewer gill rakers in outer side of first gill arch (7-8, rarely 9, vs. 8-9 to 10 in some specimens), fewer scales between lateral line and dorsal fin origin (10½-12½, vs. 13½-15½), and a faint epidermal black or violet stripe along lateral midline from eye to caudal fin base (vs. black prominent stripe). *Pseudophoxinus burduricus* is distinguished from *Pseudophoxinus maeandri* by having more lateral line and lateral series scales (21–39, vs. 19–27 and 47–57+1-2, vs. 40–44+1-2 respectively), more total vertebrae, 36-39 (vs. 35–36) (see Table 2). *Pseudophoxinus burduricus* differs from *Pseudophoxinus maeandricus* by having an incomplete lateral line (vs. complete), a shorter pelvic-fin (reaching to anus, vs. not reaching), wider and deeper head (head width at nape 55-62 % HL, vs. 46–48; head depth at nape 77–85 % HL, vs. 67–73). *Pseudophoxinus burduricus* isdistinguished from *Pseudophoxinus alii* by having more scales in the lateral series (47–57+1-2, vs. 38-43+1-2), fewer perforated scales (21–39, vs. 38–41) and smaller eyes (eye diameter 22–26 % HL, vs. 26–32). *Pseudophoxinus burduricus* is distinguished *Pseudophoxinus antalyae* by presence of a faint black or violet lateral stripe (vs. plain golden or orange stripe when alive), rounded snout (vs. pointed) and shorter pharyngeal teeth. *Pseudophoxinus burduricus* is distinguished from *Pseudophoxinus battalgilae* by an incomplete lateral line (vs. complete), fewer gill rakers in outer side of first gill arch (7–8, rarely 9, vs. 13–16), fewer branched anal fin rays (6–7, vs. 8, respectively), and the absence of a keel between the pelvic fin base and the anus (vs. presence). *Pseudophoxinus burduricus* is distinguished *Pseudophoxinus fahrettini* by an incomplete lateral line (vs. complete), fewer perforated scales lateral line (21–39, vs. 73–88) and fewer gill rakers in outer side of first gill arch (7–8, rarely 9, vs. 11–13). *Pseudophoxinus burduricus* is distinguished *Pseudophoxinus elizavetae* by having fewer scales in the lateral series (47–57+1-2, vs. 56-62+2-3), fewer gill rakers in outer side of first gill arch (7–8, rarely 9, vs. 11–13).

### Comparative material (all from Turkey)

*Pseudophoxinus alii*: IFC-ESUF 0169, 13 paratyps, 53.33–98.48 mm SL; Antalya Prov.: Ilıca Stream at Manavgat, F. Küçük, 05 May 1996.

*Pseudophoxinus antalyae*: IFC-ESUF 0159, 10, 64.07–97.10 mm SL; Antalya Prov.: Düden Canal, W.V.Neer, F. Küçük, R. Wildekamp, M. Ünlüsayın, 28 July 1996.

*Pseudophoxinus battalgilae*: IFC-ESUF 0161, 18, 46.51-109.68 mm SL; Antalya Prov.: Oymapınar Dam Lake at Manavgat, F. Küçük, 05 May 1996.

*Pseudophoxinus elizavetae*: IFC-ESUF 0174b, 10, 49.29-67.47 SL; Kayseri Prov.: Sultansazlığı, M.A. Atalay, 23 August 2004.

*Pseudophoxinus evliyae*: IFC-ESUF 0237, 26, 25.38–57.51 SL; Antalya Prov.: Kırkpınar-Korkuteli, F. Küçük, İ. Gülle, 10 May 1998. IFC-ESUF 0269, 10, 26.17–66.92 mm SL; Antalya Prov.: Kırkpınar-Korkuteli, F. Küçük, T. Şahan, 25 May 2007. -IFC-ESUF 0268, 1, 73.39 mm SL; Antalya Prov.: Kazanpınarı-Elmalı, F. Küçük, T. Şahan, 25 May 2007.

*Pseudophoxinus maeandri*: IFC-ESUF 0248, 8, 46.70–55.96 mm SL; Denizli Prov.: Lake Işıklı source, F. Küçük, M.A. Atalay, N. Bogutskaya & A.Naseka, 14 August 2006.

*Pseudophoxinus maeandricus*: 3, 60.71–78.70 mm SL; Afyon Prov.: Karadirek Stream-Sandıklı, V. Yeğen, 29 June 2006.

*Pseudophoxinus ninae*: IFC-ESUF 0263, 4, 48.9–67.5 mm SL; Burdur Prov.: Pınargözü-Bucak, F. Küçük, T. Şahan, 25 May 2007. –IUSHM 33900-928, 15, 46.4–68.4 mm SL; Burdur Prov.: Onaç Stream, M. Özuluğ, J. Freyhof, 12 June 2006.

## Supplementary Material

XML Treatment for Pseudophoxinus burduricus
